# Common and Specific Mechanisms of Desiccation Tolerance in Two Gesneriaceae Resurrection Plants. Multiomics Evidences

**DOI:** 10.3389/fpls.2019.01067

**Published:** 2019-09-04

**Authors:** Jie Liu, Daniela Moyankova, Dimitar Djilianov, Xin Deng

**Affiliations:** ^1^Facility Horticulture Laboratory of Universities in Shandong, Weifang University of Science and Technology, Shouguang, China; ^2^Key Laboratory of Plant Resource, Institute of Botany, Chinese Academy of Sciences, Beijing, China; ^3^Abiotic Stress Group, Agrobioinstitute, Agricultural Academy, Sofia, Bulgaria

**Keywords:** *Boea hygrometrica*, *Haberlea rhodopensis*, desiccation tolerance, transcriptome, metabolome, Gesneriaceae, resurrection plant

## Abstract

Environmental stress, especially water deficiency, seriously limits plant distribution and crop production worldwide. A small group of vascular angiosperm plants termed “resurrection plants,” possess desiccation tolerance (DT) to withstand dehydration and to recover fully upon rehydration. In recent years, with the rapid development of life science in plants different omics technologies have been widely applied in resurrection plants to study DT. *Boea hygrometrica* is native in East and Southeast Asia, and *Haberlea rhodopensis* is endemic to the Balkans in Europe. They are both resurrection pants from Gesneriaceae family. This paper reviews recent advances in transcriptome and metabolome, and discusses the differences and similarities of DT features between both species. Finally, we believe we provide novel insights into understanding the mechanisms underlying the acquisition and evolution of desiccation tolerance of the resurrection plants that could substantially contribute to develop new approaches for agriculture to overcome water deficiency in future.

## Introduction

Water is of crucial importance for the life on our planet. Water availability affects plants distribution in nature and crop yields in cultivated areas. Most plants encounter water stress at certain stages of their life cycles. In order to survive, plants have evolved various mechanisms and adaptation strategies. For example, plants enhance their water-holding capacity through stomatal regulation and leaf specific structure, enhance water-absorbing capacity by promoting root growth, improve osmotic regulation capacity by accumulating osmoprotectants, and reduce oxidative damages by regulation of scavenging enzymes and agents. Although these mechanisms are usually effective against moderate drought stresses, they can’t help plants to cope with severe and persistent desiccation.

Desiccation tolerance (DT) in angiosperm vegetative tissues is a rare phenomenon—only a small group of vascular plants termed “resurrection plants,” evolved uniquely to tolerate the loss of more than 90% of their water content, and to recover rapidly after rehydration (Moore et al., 2009; Dinakar et al., 2012; Gaff and Oliver, 2013). About 300 species have been reported to tolerate severe desiccation. Geographically, they are distributed predominantly in the southern hemisphere—in Africa, America, and Australia (Alpert and Oliver, 2002; Gaff and Oliver, 2013). However, DT species could be found in the northern hemisphere, too. Interestingly enough and with lack of sufficient scientifically sound explanations so far, the only resurrection angiosperm plants, found in Europe and Asia belong to one and the same family—Gesneriaceae (Shen et al., 2004; Huang et al., 2012; Mitra et al., 2013; Li et al., 2014; Djilianov et al., 2016; Zhang et al., 2016). The resurrection species in Europe—*Haberlea rhodopensis*, *Ramonda myconii*, *R. serbica*, and *R. nathaliae* are phylogenetically relatively close and live in more or less similar environments (Petrova et al., 2015; Rakić et al., 2015; Fernández-Marín et al., 2018). The Asia endemic resurrection species, including *Boea hygrometrica* (also named *Dorcoeras hygrometricum*), *B. clarkeana*, *B. crassifolia*, *Paraboea rufescens*, and *Paraisometrum mileense*, are phylogenetically separated from European clade according to molecular phylogenetic analyses (Shen et al., 2004; Wang et al., 2010; Huang et al., 2012; Mitra et al., 2013; Li et al., 2014; Djilianov et al., 2016; Zhang et al., 2016).

Recent advances in genome sequencing and various “omics” technologies have greatly facilitated the discovery of DT-related genes and improved the understanding of DT molecular mechanisms. Transcriptome and metabolome studies were also performed in *B. hygrometrica* (Xiao et al., 2015; Zhu et al., 2015; Sun et al., 2018) and *H. rhodopensis* (Gechev et al., 2013; Moyankova et al., 2014; Mladenov et al., 2015; Liu et al., 2018). *B. hygrometrica* is distributed in East and Southeast Asia, and *H. rhodopensis* is endemic to the Balkans in Europe (Mitra et al., 2013; Petrova et al., 2015). They both prefer shady slopes and limestone rocks and are subjected to frequent cycles of drying and rehydration under natural conditions, but spend dry state in different seasons. *B. hygrometrica* remains desiccated during winter while *H. rhodopensis* withstands desiccation during the dry periods of warm Mediterranean summer. Thus, it would be interesting to know if their DT mechanisms share common features and origin. Although evidences are still limited, this review will elaborate on the existing data in an attempt to better understand the common and specific mechanisms underlying DT of these Gesneriaceae resurrection plants. 

## Photosynthesis

Photosynthesis is the most important photo-chemical reaction in plants, and it is seriously disrupted by dehydration (Pinheiro and Chaves, 2011). Like other homoiochlorophllous DT species (including the *Ramonda spp.*), *B. hygrometrica* and *H. rhodopensis* can keep their photosynthetic apparatus intact, maintain chlorophyll pigments–protein complexes stable, retain major pigment contents, and employ conserved and novel antioxidant enzymes/metabolites to minimize the possible oxidative damage (Deng et al., 2003; Georgieva et al., 2005; Georgieva et al., 2007; Georgieva et al., 2009; Giarola et al., 2017). In parallel with the above studies, transcriptome data and protein studies showed that the expression of the major photosynthesis-related genes and proteins related to the subunits of photosystem I (PS I) and photosystem II (PS II), light-harvesting complexes II and I, cytochrome b6/f complex and electron transport were down-regulated, while many other chloroplastic genes and proteins were up-regulated to protect the system during dehydration in both *B. hygrometrica* and *H. rhodopensis* (Jiang et al., 2007; Sarvari et al., 2014; Xiao et al., 2015; Liu et al., 2018). For example, early light-inducible proteins (ELIPs) transcripts were up-regulated in response to dehydration (Gechev et al., 2013; Xiao et al., 2015) to protect the photosynthetic machinery from photooxidative damage by preventing the accumulation of free chlorophyll, binding pigments and preserving the chlorophyll–protein complexes. Combining prompt fluorescence and adsorption spectroscopy, in *H. rhodopensis* we found that the cyclic electron flow (CEF) and PSII maintain significant activity till severe dehydration stage—opposite to the steep decrease of linear electron flow (LEF) (Mladenov et al., 2015). Transcriptome data showed that ferredoxin and NADPH-quinone oxidoreductase subunit genes are up-regulated during dehydration (Liu et al., 2018). Together, these data suggest that CEF is activated to generate more ATP and balance the ATP/NAPDH ratio in dehydrated *H. rhodopensis*. Similarly, in *B. hygrometrica* CEF around PS I is maintained during moderate dehydration, however, LEF around PS II was found to be dramatically decreased (Tan et al., 2017).

## Protective Proteins

Dehydration impacts cellular functions and viability, compromising the macromolecular structures—in particular, the membranes and proteins (Garrido et al., 2012). Late embryogenesis abundant (LEA) and heat shock proteins (HSPs) are two major classes of protective proteins. Various roles have been attributed to LEA proteins: stabilization and protection of proteins, nucleic acids, and cell membranes, decrease of reactive oxygen species (ROS) and ions, and contribution together with sugars for glassy state formation (Tunnacliffe et al., 2010). LEA genes have been reported to be induced during dehydration in many resurrection plants including *B. hygrometrica* and *H. rhodopensis* (Liu et al., 2009; Rodriguez et al., 2010; Xiao et al., 2015; Liu et al., 2018; Liu et al., 2019). Furthermore, LEA genes (*BhLEA1* and *BhLEA2*) from *B. hygrometrica* have been shown to contribute to protein stabilization in drought-stressed transgenic tobacco plants thus improving their tolerance (Liu et al., 2009).

HSPs are a class of molecular chaperones that bind to and prevent aggregation of unfolded proteins and also help in maintaining protein structure under denaturing conditions, such as desiccation. Transcripts that encode HSP70s, DNAJs/HSP40s, sHSPs and heat shock factors (HSFs) accumulate to high levels during dehydration in *B. hygrometrica* and *H. rhodopensis* (Gechev et al., 2013; Zhang et al., 2013; Zhu et al., 2015; Liu et al., 2018). BhDNAJC2 from *B. hygrometrica* is the first and, so far, the only reported dehydration-inducible DNAJ/HSP40 protein in resurrection plants, and it plays a significant role in plant resistance to stresses, as shown in transgenic *Arabidopsis* plants (Chen et al., 2015). Interestingly enough, some of *H. rhodopensis* LEAs and HSPs were abundant under well-watered conditions, suggesting that the transcriptome and proteome of this plant species may be primed to some extent for DT (Gechev et al., 2013).

## Protein-Quality Control

Protein homeostasis is central for normal cellular function, but often disturbed under abiotic stress conditions. Protein quality control systems recruit specific factors and pathways to regulate protein refolding or misfolded proteins degradation in the endoplasmic reticulum (ER), cytosol, and other cell compartments (Ron and Walter, 2007). Transcriptomic data revealed that protein folding was enriched among up-regulated genes in desiccated *B. hygrometrica* and at the same time, marker genes of unfolded protein stress in the ER and chaperone genes in cytosolic protein quality control were induced by dehydration (Zhu et al., 2015). Furthermore, a key regulator of ER quality control, bZIP60 was activated via mRNA alternative splicing during dehydration (Wang et al., 2017). Similarly, in *H. rhodopensis*, two key regulators [ER lumen protein-retaining receptor 2 (ERD2) and ER oxidoreductin-1 (ERO1)] were up-regulated in late stage of dehydration, reflecting the activation of unfolded protein response to mitigate drought-induced protein-unfolding (Liu et al., 2018). These are the only two reports of protein-quality control in resurrection plants so far, but they could be a good background for elucidation of this process, to uncover their fundamental role in conferring DT in other species.

## Autophagy

Autophagy is a major form of programmed cell death but also plays an important role in anti-cell death, facilitating precursor recycling and energy supply under stressed conditions (Patel et al., 2006). It also serves as one of the two major mechanisms of protein degradation (Yue and Calderwood, 2011). Autophagy was initially found to play a major role in desiccation tolerance in yeast and the resurrection grass *Tripogon loliiformis* (Ratnakumar et al., 2011; Williams, 2015). Similarly, we have confirmed that autophagy genes were also enriched among desiccation-induced transcripts in *B. hygrometrica* and *H. rhodopensis* (Zhu et al., 2015; Liu et al., 2018). These observations suggest a common role of autophagy for DT in both yeast and plants.

## DNA Repair

Transcripts related to DNA repair were found to be induced during dehydration only in *H. rhodopensis* (Liu et al., 2018). Especially, the genes of nucleotide excision repair (NER) pathway were activated, indicating the importance of DNA repair to maintain genome integrity during severe dehydration. No NER related transcripts with significantly changed expression levels were found in *B. hygromtrica* transcriptomic data (Xiao et al., 2015; Zhu et al., 2015). There is no report on the activation of NER under desiccation in other resurrection plants so far, thus the potential role of NER for DT needs to be further studied.

## Primary and Secondary Metabolites

Accumulation of soluble sugars under stress is a common feature and resurrection plants make no exception. Sucrose is considered the most accumulated sugar in higher resurrection plants (Ingram and Bartels, 1996; Oliver et al., 2010), possibly acting as a water replacement molecule which contributes in membrane and protein structure stabilization or cytoplasm vitrification of desiccated tissue, and as potential energy supply during rehydration (Abdalla et al., 2010; Vicré et al., 2010; Griffiths et al., 2014). In our case, *H. rhodopensis* and *B. hygrometrica* plants increase the accumulation of sucrose during desiccation, same as *Ramonda spp.* and many other resurrection plants (Müller et al., 1997; Whittaker et al., 2001; Peters et al., 2007; Djilianov et al., 2011; Dinakar and Bartels, 2013; Gechev et al., 2013; Moyankova et al., 2014; Mladenov et al., 2015; Sun et al., 2018).

In addition to the significant role of sucrose, it is important to outline the role of raffinose in *H. rhodopensis and B. hygrometrica*, in conferring cellular protection and preventing crystallization of sucrose during drying, as in many other DT angiosperms (Müller et al., 1997; Farrant et al., 2007). In *H. rhodopensis*, *R. nathaliae* and *R. myconi*, the level of this compound is high at normal conditions and only slightly changed in desiccated plants, suggesting its potential role for priming the plant stress response (Müller et al., 1997; Djilianov et al., 2011; Gechev et al., 2013; Moyankova et al., 2014). On the other hand, in *B. hygrometrica*, raffinose family oligosaccharides accumulate significantly and genes related to their biosynthesis were induced during dehydration (Wang et al., 2009; Wang et al., 2012), which coincide with the need of priming for DT in this plant.

Apart from those primary metabolites, many secondary metabolites, especially antioxidants, were found to change during dehydration in many resurrection plants including *B. hygrometrica* and *H. rhodopensis* (Moyankova et al., 2014; Sun et al., 2018). For example, α-tocopherol, the lipid soluble antioxidant that protects plant cells against oxidative stresses, was increased at fully desiccated stage in both *H. rhodopensis* and *B. hygrometrica* as in other resurrection plants—e.g. *Sporobolus stapfianus* (Oliver et al., 2011). In agreement with the metabolomics data, transcripts encoding enzymes in α-tocopherol biosynthesis including homogentisate phytyltransgerase1 (HPT1) and tocopherol cyclase (VTE1) were up-regulated during dehydration both in *B. hygrometrica* and *H. rhodopensis*. (Zhu et al., 2015; Liu et al., 2018).

Several metabolites have been detected to change their abundance in response to dehydration in *B. hygrometrica* so far. The ascorbic acid content increased to high levels in rehydrated plants of *B. hygrometrica*, albeit the expression patterns of genes related to ascorbic acid metabolism and oxidation–reduction are complex (Zhu et al., 2015). Glutaric acid accumulation was also in line with desiccation tolerance in *B. hygrometrica* (Sun et al., 2018). The accumulation of these metabolites is positively correlated with the transcript abundance of the related genes. Furthermore, the global unbiased metabolomics combined with the microarray data analysis also suggests a role of putative regulators involved in ubiquitination and ABA signal transduction in the acquisition of rapid desiccation tolerance in *B. hygrometrica* (Sun et al., 2018). On the other hand, the glycoside myconoside and some phenolic acids were high under normal conditions and indicated their importance for priming of *H. rhodopensis* to withstand severe desiccation (Moyankova et al., 2014). Total phenols, glutathione, *β*-aminoisobutyric acid, *β*-sitosterol increased up to several times at later stages of desiccation of *H. rhodopensis* (Djilianov et al., 2011; Moyankova et al., 2014). These difference highlights the possibility of species-specific regulation of metabolism, although it is not clear whether all of them are involved in DT mechanism.

Studies on endogenous plant hormones in *H. rhodopensis* (Djilianov et al., 2013; Liu et al., 2018) showed that jasmonic acid, along and even earlier than abscisic acid (ABA), provide a triggering signal to unlock the response to desiccation. High levels of salicylic acid, early stages’ accumulation of ethylene and dynamic changes of cytokinins and auxins actively participate in the dehydration response. Most of the genes, associated with the metabolism of the list and other compounds have been found to be a subject of reprograming at the late stage of desiccation that defines the DT in *H. rhodopensis* (Liu et al., 2018). In *B. hygrometrica*, KEGG pathways of biosynthesis of other secondary metabolites and biosynthesis of plant hormones and GO terms of jasmonic acid biosynthetic process, ethylene biosynthetic process and abscisic acid mediated signaling are enriched among differentially expressed genes in response to dehydration (Zhu et al., 2015), although the composition of secondary metabolites and contents of hormones has not been studied in detail.

## Conclusion and Future Perspectives

**Figure 1:**
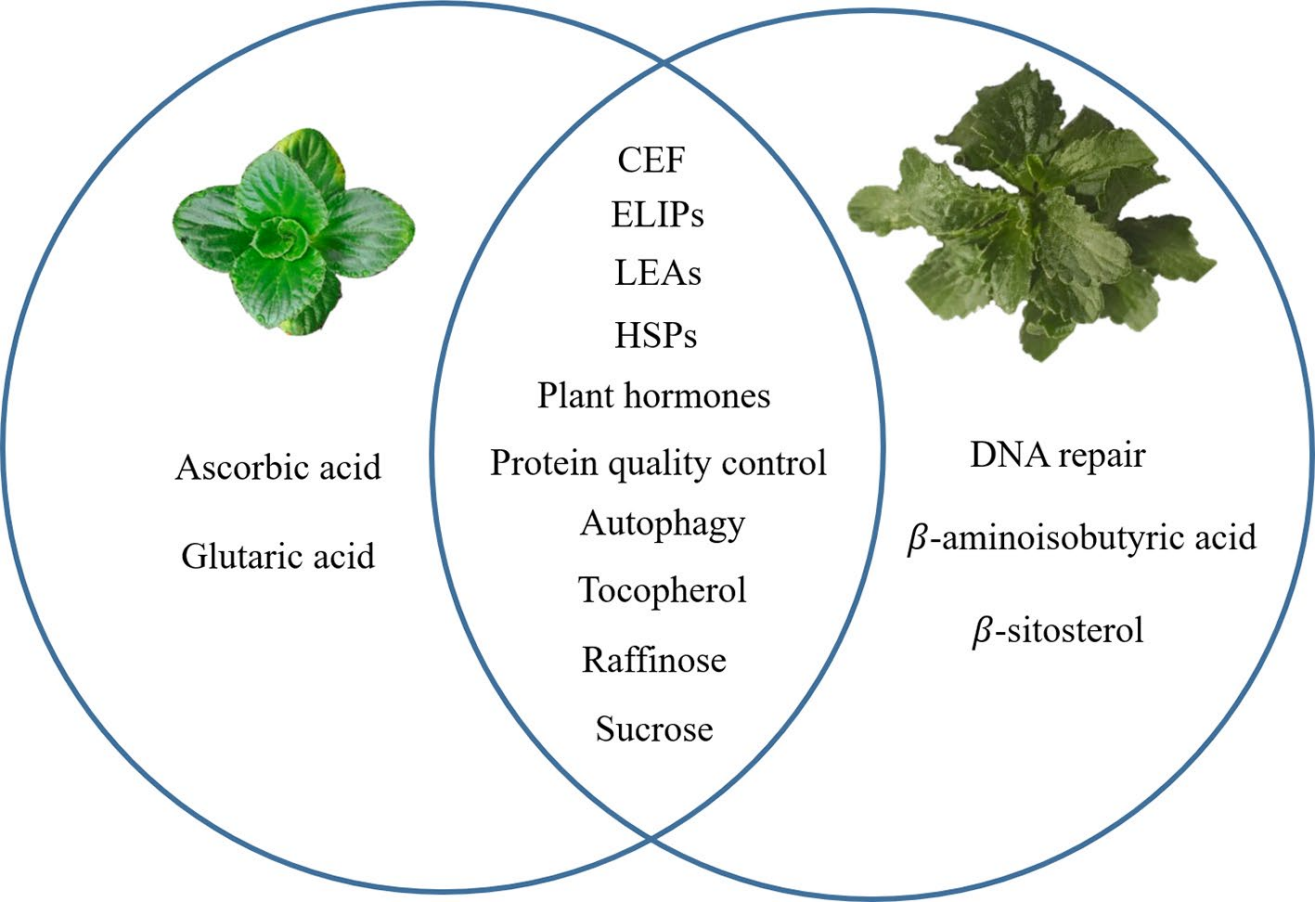
A graphical summary for common and specific mechanisms of desiccation tolerance in *Boea hygrometrica* and *Haberlea rhodopensis*. The left circle represents the specific mechanisms of *B. hygrometrica*; the right circle represents the specific mechanisms of *H. rhodopensis*; the intersection of the two circles represents common mechanisms.

Recent progress in transcriptomes and metabolomes allowed us to compare the DT between *B. hygrometrica* and *H. rhodopensis*. When similar mechanisms were studied in both species, common responses include LEA and HSPs proteins, protein quality control, sugar metabolism, α-tocopherol accumulation, autophagy and plant hormone metabolism and signaling. Specific features such as ascorbic acid and glutaric acid accumulation at desiccation were found only in *B. hygrometrica*, while DNA repair and increase of β-aminoisobutyric acid and β-sitosterol—in *H. rhodopensis* (**Figure 1**). These data could infer the highly complex mechanisms that contribute to the ability of Gesneriaceae resurrection plants to withstand desiccation. Even in one botanical family, the DT has been independently evolved in these species, probably due to their diverse phylogeographic history.

## Author Contributions

XD designed the frame of the manuscript. JL wrote the first draft of the manuscript. DM wrote sections of the manuscript. DD and XD revised the manuscript. All authors contributed to manuscript revision and read and approved the submitted version.

## Funding

This work was supported by National Natural Science Foundation of China (No.31600155), Scientific Research Project of Facility Horticulture Laboratory of Universities in Shandong (No. 2018YY034), China-Bulgari’s Inter-Governmental S&T Cooperation Project [14-10], and Bulgarian National Science Fund (DNTS/China/01/7).

## Conflict of Interest Statement

The authors declare that the research was conducted in the absence of any commercial or financial relationships that could be construed as a potential conflict of interest.
